# Cellular Source-Specific Effects of Apolipoprotein (Apo) E4 on Dendrite Arborization and Dendritic Spine Development

**DOI:** 10.1371/journal.pone.0059478

**Published:** 2013-03-19

**Authors:** Sachi Jain, Seo Yeon Yoon, Laura Leung, Johanna Knoferle, Yadong Huang

**Affiliations:** 1 Gladstone Institute of Neurological Disease, San Francisco, California, United States of America; 2 Gladstone Institute of Cardiovascular Disease, San Francisco, California, United States of America; 3 Biomedical Sciences Graduate Program, University of California San Francisco, California, United States of America; 4 Department of Neurology, University of California San Francisco, California, United States of America; 5 Department of Pathology, University of California San Francisco, California, United States of America; National Center for Scientific Research Demokritos, Greece

## Abstract

Apolipoprotein (apo) E4 is the leading genetic risk factor for Alzheimer’s disease (AD), and it has a gene dose-dependent effect on the risk and age of onset of AD. Although apoE4 is primarily produced by astrocytes in the brain, neurons can also produce apoE4 under stress conditions. ApoE4 is known to inhibit neurite outgrowth and spine development *in vitro* and *in vivo*, but the potential influence of apoE4’s cellular source on dendritic arborization and spine development has not yet been investigated. In this study, we report impairments in dendritic arborization and a loss of spines, especially thin (learning) and mushroom (memory) spines, in the hippocampus and entorhinal cortex of 19–21-month-old female neuron-specific-enolase (NSE)-apoE4 and apoE4-knockin (KI) mice compared to their respective apoE3-expressing counterparts. In general, NSE-apoE4 mice had more severe and widespread deficits in dendritic arborization as well as spine density and morphology than apoE4-KI mice. The loss of dendritic spines, especially mushroom spines, occurred in NSE-apoE4 mice as early as 7–8 months of age. In contrast, glial fibrillary acidic protein (GFAP)-apoE4 mice, which express apoE4 solely in astrocytes, did not have impairments in their dendrite arborization or spine density and morphology compared to GFAP-apoE3 mice at both ages. These results indicate that the effects of apoE4 on dendrite arborization, spine density, and spine morphology depend critically on its cellular source, with neuronal apoE4 having more detrimental effects than astrocytic apoE4.

## Introduction

Apolipoprotein (apo) E is a 299-amino-acid glycoprotein that is highly expressed in the mammalian brain and occurs as three isoforms in humans–apoE2, apoE3, and apoE4. Although these isoforms differ from each other by only one or two amino acid substitutions at positions 112 and 158, they have profound functional differences in relation to neurological disorders [1], [2], [3], [4]. ApoE4 is the major genetic risk factor for Alzheimer’s disease (AD) [5], [6], with a gene dose-dependent effect on the risk and age of disease onset. Individuals with one copy of the apoE4 gene have a 45% chance of developing AD by age 85, which increases to 50–90% for individuals with two copies [7], [8].

ApoE is primarily synthesized by astrocytes in the brain, but it can also be made by neurons under stress conditions [3], [9], [10], [11]. The cellular source of apoE4 influences its pathological effects *in vitro* and *in vivo.* For instance, neuronal – but not astrocytic – apoE4 undergoes proteolytic cleavage, resulting in C-terminally truncated fragments that cause tau phosphorylation, mitochondrial impairments, and neurodegeneration [3], [12], [13], [14]. Neuronal apoE4 also increases susceptibility to excitotoxin-induced cell death compared to neuronal apoE3, whereas astrocytic apoE3 and apoE4 are equally excitoprotective [15]. Neuron-specific-enolase (NSE)-apoE4 mice, which express human apoE4 solely in neurons, exhibit spatial learning and memory deficits compared to NSE-apoE3 mice [16]. ApoE4-knockin (KI) mice, which express apoE4 under the control of an endogenous apoE promoter, are also impaired in spatial learning and memory, albeit at an older age [17]. In contrast, mice whose apoE4 expression is limited to astrocytes do not show spatial learning and memory deficits, although they do show impairments in working memory [18].

Dendritic pathology and the loss of dendritic spines and synapses are prominent features in early-stage AD [19], [20]. ApoE4-associated abnormalities in dendrite arborization and spine development were reported in both primary neuronal cultures and in transgenic mice [21], [22], [23], [24], [25], [26], which has been suggested to be linked to apoE4- and its fragment-induced mitochondrial impairments and an inherited variable ploy-T repeat genotype in the mitochondrial out-membrane protein TOMM40 [3], [24], [27], [28]. However, whether these effects are influenced by the cellular source of apoE4 has not yet been investigated. Here, we report impaired dendrite arborization and decreased total, thin, and mushroom spine densities in female apoE4-KI and NSE-apoE4 mice compared to their apoE3 counterparts in both the hippocampus and the entorhinal cortex at 19–21 months of age. In contrast, the dendritic arborization and spine density of glial fibrillary acidic protein (GFAP)-apoE4 mice – which express apoE4 solely in astrocytes – were not impaired compared to GFAP-apoE3 mice. These results indicate that the cellular source of apoE4 profoundly influences its effects on neuronal structure.

## Materials and Methods

### Transgenic Mice

We analyzed transgenic mice expressing human apoE3 or apoE4 in CNS neurons under control of a neuron-specific enolase promoter on a mouse apoE-deficient background [15], [16], [29]. All mice were backcrossed >20 times with apoE-deficient mice (C57BL/6J-Apoetm-1Unc; The Jackson Laboratory, Bar Harbor, ME). Transgenic mice expressing human apoE3 or apoE4 driven by a glial fibrillary acidic protein promoter (backcrossed >10 times) were generated on a mouse apoE-deficient background as described [13], [15]. Human apoE3- or apoE4-KI mice, in which the mouse apoE gene is replaced by the human apoE sequence [30], were from Taconic. All animal procedures were approved by the Gladstone Institutes and the University of California San Francisco Animal Care and Use Committees.

### Golgi Staining

Golgi staining was performed on all mouse genotypes at 7–8 and 19–21 months of age (n = 3–5 mice/genotype). For these experiments, we used the FD Rapid Golgi Stain kit (FD NeuroTechnologies, Ellicott City, MD) as described previously [26], [31]. Briefly, freshly dissected brains were immersed in equal parts solution A and B for 2 weeks at room temperature, then transferred to solution C for 4 days at 4°C. Brains were sliced at a thickness of 150 µm using a freezing-sliding microtome (Leica SM2000R).

### Analyses of Dendrite Arborization and Dendritic Spine Density and Morphology

To analyze dendrite length and number, brightfield images of individual pyramidal neurons were taken with a Leica CTR5000 10X objective. Apical and basal dendrites were traced using the NeuronJ plugin of ImageJ [32], and total dendrite length and number were recorded. For analysis of dendritic spine density and morphology, the apical and basal dendrites of pyramidal neurons from the CA1 region of the hippocampus, entorhinal cortex, and auditory cortex were imaged with a Leica CTR5000 brightfield 63X oil objective. Protrusions were classified into one of four subtypes–stubby, thin, mushroom, or filopodia–based on published criteria [31], [33]. Images were coded and analyzed in a blinded manner using ImageJ software [24], [31], [34].

### Statistical Analysis

All values are expressed as mean ± SEM. Statistical analyses were performed with GraphPad Prism software. Differences between the means were assessed by *t*-test or one-way ANOVA. A *p*-value of <0.05 was considered to be statistically significant. Statistical values are denoted as follows: *p<0.05, **p<0.01, ***p<0.001.

## Results

### The Cellular Source of apoE4 Determines its Effects on Dendritic Arborization

We examined dendritic arborization in several apoE4 mouse models and their apoE3-expressing controls; namely: NSE-apoE4 (versus NSE-apoE3), apoE4-KI (versus apoE3-KI), and GFAP-apoE4 (versus GFAP-apoE3). Since some of these apoE4 mice did not develop spatial learning and memory deficits until 16–18 months [17], we first analyzed these mice at 19–21 months of age. Only female mice were used due to their enhanced susceptibility to apoE4-induced learning and memory deficits [16], [17], [35]. Pyramidal neurons from the CA1 region of the hippocampus, the entorhinal cortex, and the auditory cortex were analyzed using the rapid Golgi-Cox impregnation method. Apical oblique (AO) and basal shaft (BS) dendrites were examined separately.

**Figure 1:**
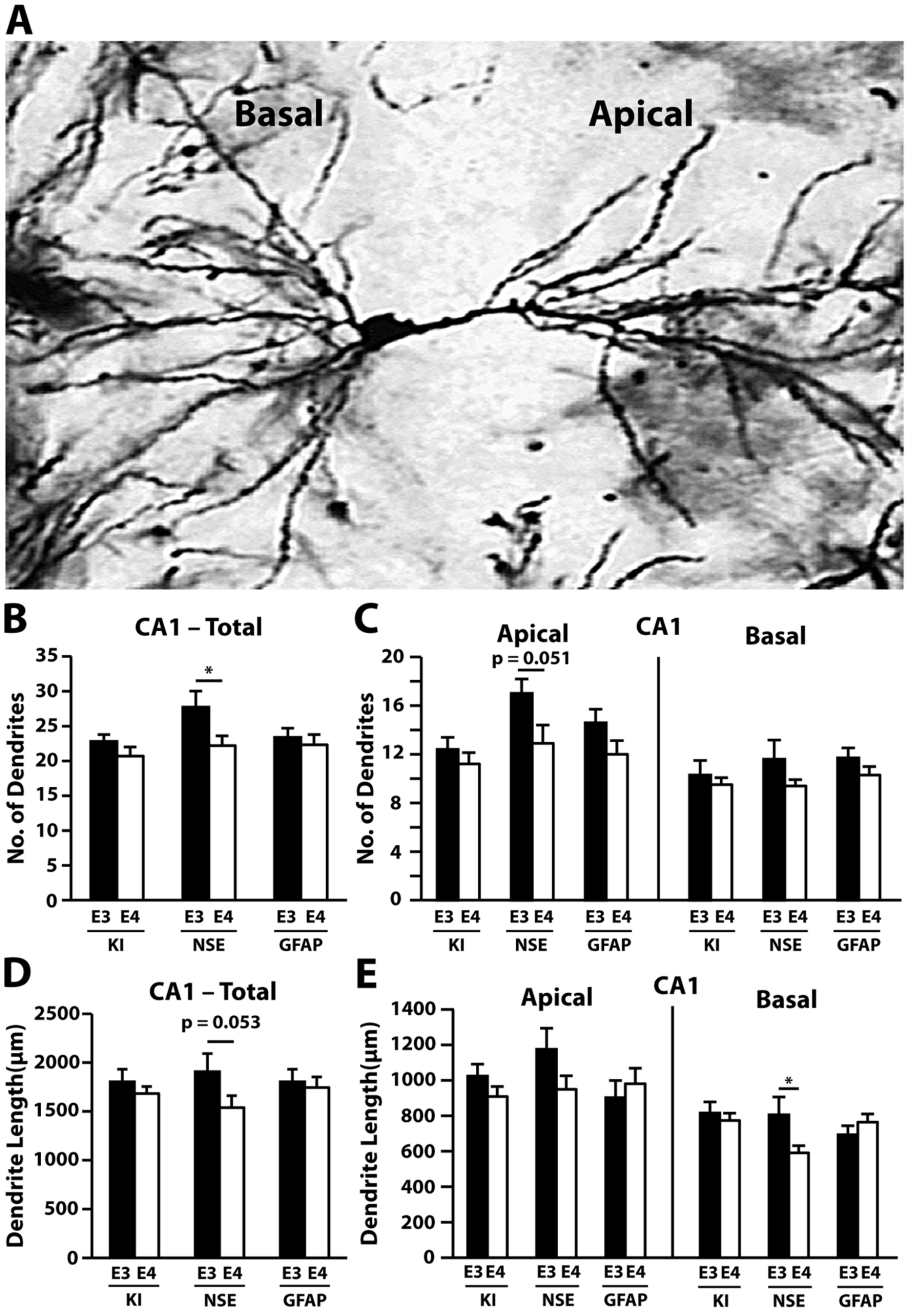
Cellular source of apoE influences dendrite number and length in the CA1 region of the hippocampus at 19–21 months of age. (*A*) A representative Golgi-stained image of a CA1 pyramidal neuron. (*B*) Quantification of the total number of CA1 dendrites, combining AO+BS dendrites. (*C*) Number of AO and BS dendrites in the CA1. (*D*) Quantification of the total length of CA1 dendrites, combining AO+BS dendrites. (*E*) Length of AO and BS dendrites in the CA1. Values are mean ± SEM, n = 13–15 neurons from 3–5 mice for each group. *p<0.05.

We first measured the total length and number of CA1 dendrites in NSE-apoE4, apoE4-KI, and GFAP-apoE4 mice and their respective apoE3 counterparts (Fig. 1A). Quantitative analysis revealed that in the CA1, the total number of dendrites in NSE-apoE3 mice was higher than in NSE-apoE4 mice (Fig. 1B). Further analysis revealed a trend toward a greater number of apical dendrites in NSE-apoE3 mice than in NSE-apoE4 mice in the CA1, although this difference did not reach statistical significance (Fig. 1C). NSE-apoE4 mice showed a trend toward decreased total dendrite length in the CA1 (Fig. 1D) compared to NSE-apoE3 mice, and basal NSE-apoE4 dendrites were significantly shorter than basal NSE-apoE3 dendrites (Fig. 1E). One-way ANOVA revealed significantly shorter basal dendritic length in NSE-apoE4 mice than in apoE4-KI and GFAP-apoE4 mice (Fig. 1E). Neither apoE4-KI nor GFAP-apoE4 mice showed significant differences in dendrite number (Fig. 1B) or length (Fig. 1D) compared to their apoE3 controls.

**Figure 2:**
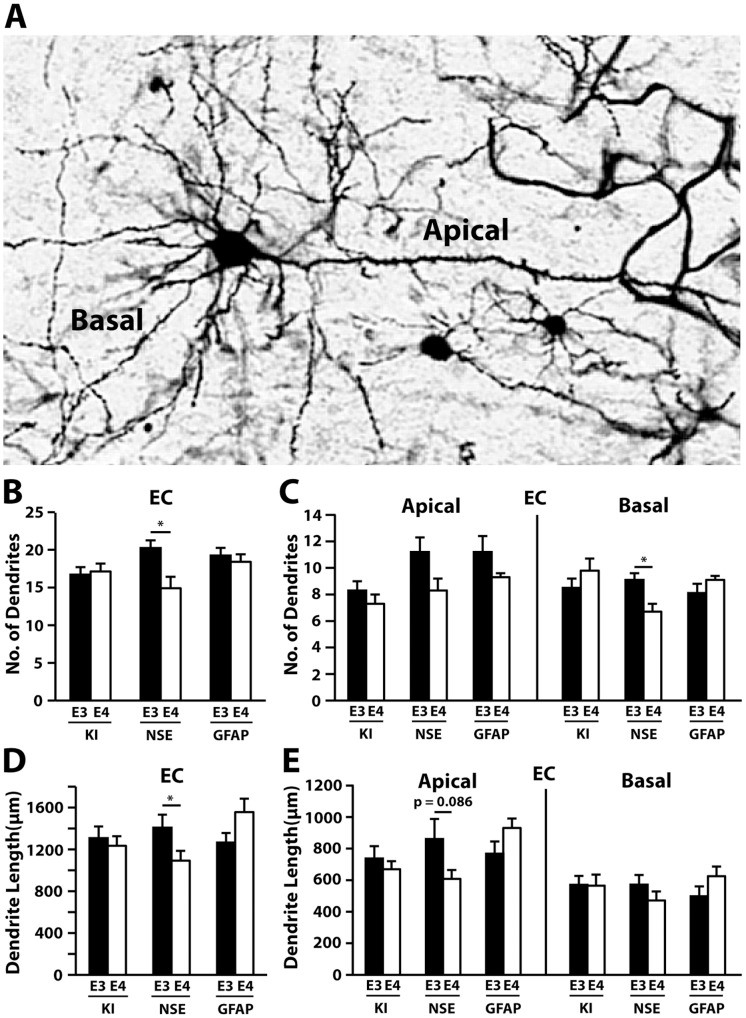
Cellular source of apoE influences dendrite number and length in the entorhinal cortex at 19–21 months of age. (*A*) A representative Golgi-stained image of a pyramidal neuron from the entorhinal cortex. (*B*) Quantification of the total number of entorhinal cortex dendrites, combining AO+BS dendrites. (*C*) Number of AO and BS dendrites in the entorhinal cortex. (*D*) Quantification of the total length of entorhinal cortex dendrites, combining AO+BS dendrites. (*E*) Length of AO and BS dendrites in the entorhinal cortex. Values are mean ± SEM, n = 13–15 neurons from 3–5 mice for each group. *p<0.05.

**Figure 3:**
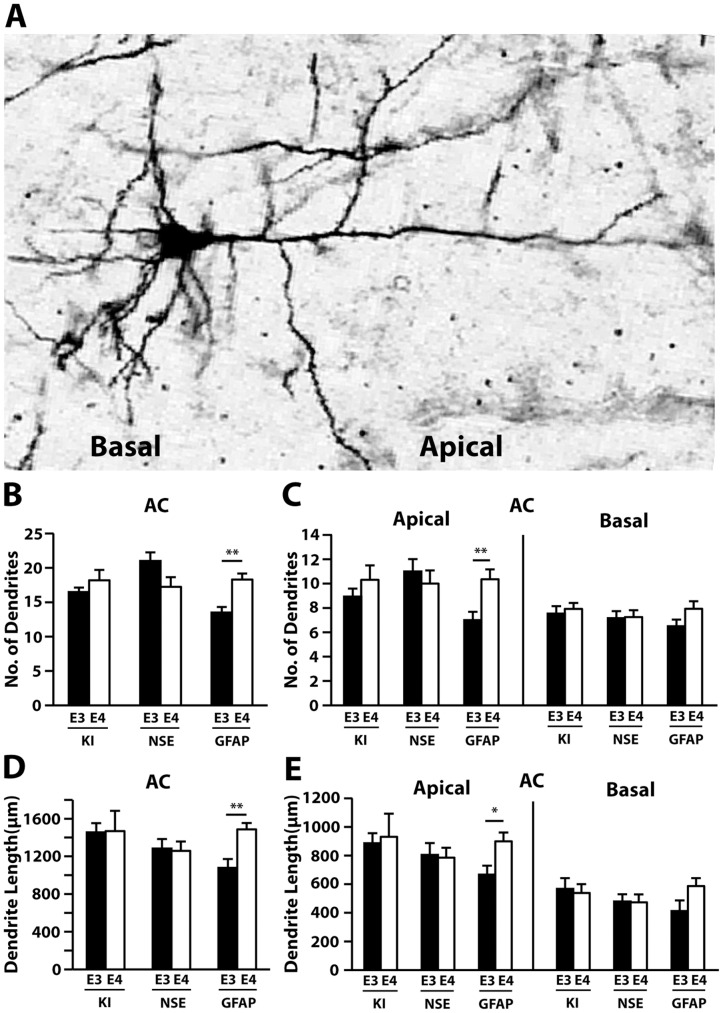
Cellular source of apoE influences dendrite number and length in the auditory cortex at 19–21 months of age. (*A*) A representative Golgi-stained image of a pyramidal neuron from the auditory cortex. (*B*) Quantification of the total number of auditory cortex dendrites, combining AO+BS dendrites. (*C*) Number of AO and BS dendrites in the auditory cortex. (*D*) Quantification of the total length of auditory cortex dendrites, combining AO+BS dendrites. (*E*) Number of AO and BS dendrites in the auditory cortex. Values are mean ± SEM, n = 13–15 neurons from 3–5 mice for each group. *p<0.05, **p<0.01.

As in the CA1, NSE-apoE3 mice had a greater number of dendrites in the entorhinal cortex than NSE-apoE4 mice (Fig. 2A,B), and this difference reflected changes in basal dendrite numbers (Fig. 2C). A reduction in total dendritic length was also observed in NSE-apoE4 mice (Fig. 2D). In contrast, apoE4-KI and GFAP-apoE4 mice had similar dendritic numbers (Fig. 2B) and lengths (Fig. 2D) compared to their apoE3 controls in the entorhinal cortex. When analyzed separately, apical, but not basal, dendrites showed a trend toward shorter length in NSE-apoE4 mice compared to NSE-apoE3 mice (Fig. 2E). In the auditory cortex (Fig. 3A), GFAP-apoE4 mice showed an increase in total dendritic number (Fig. 3B) and length (Fig. 3D) compared to GFAP-apoE3 mice, which reflected changes in apical, but not basal, dendrite number (Fig. 3C,E). However, neither apoE4-KI nor NSE-apoE4 mice showed differences in dendrite number or length compared to their apoE3 controls in the auditory cortex (Fig. 3B,D).

### The Cellular Source of apoE4 Determines its Effects on Dendritic Spine Density

**Figure 4:**
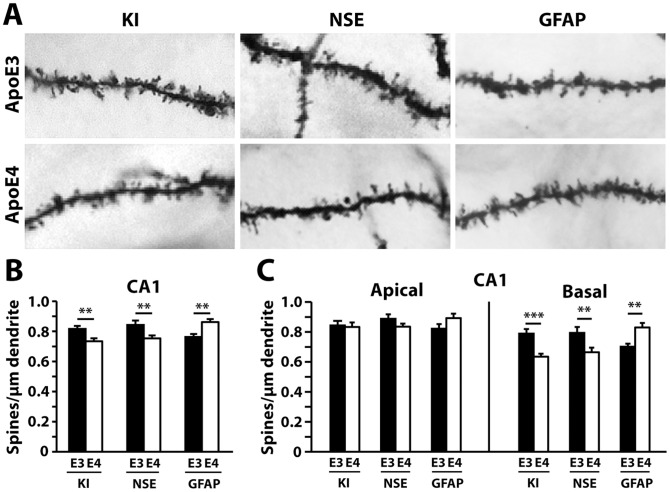
NSE-apoE4 and apoE4-KI, but not GFAP-apoE4, mice have reduced dendritic spine density in the CA1 region compared to apoE3 counterparts at 19–21 months of age. (*A*) Representative images of dendrites with spines from the CA1 region of NSE-apoE3, NSE-apoE4, apoE3-KI, apoE4-KI, GFAP-apoE3, or GFAP-apoE4 mouse brains. (*B*) Quantification of total spine density in the CA1 region, combining AO+BS spines. (*C*) Number of AO and BS spines in the CA1. Values are mean ± SEM, n = 28–33 neurons from 3–5 mice for each group. **p<0.01, ***p<0.001.

We next examined whether the cellular source of apoE4 might differentially impact dendritic spine density. In the CA1 region, both NSE-apoE4 and apoE4-KI mice had significant reductions in total spine number compared to their apoE3 controls. Surprisingly, GFAP-apoE4 mice had more spines compared to GFAP-apoE3 mice in the CA1 region (Fig. 4A,B). Moreover, these differences exclusively reflected alterations in basal spines, with the apical spine densities showing no significant differences (Fig. 4C). One-way ANOVA revealed significant reductions in total and basal spines in NSE-apoE4 and apoE4-KI mice compared to GFAP-apoE4 mice (Fig. 4B,C).

**Figure 5:**
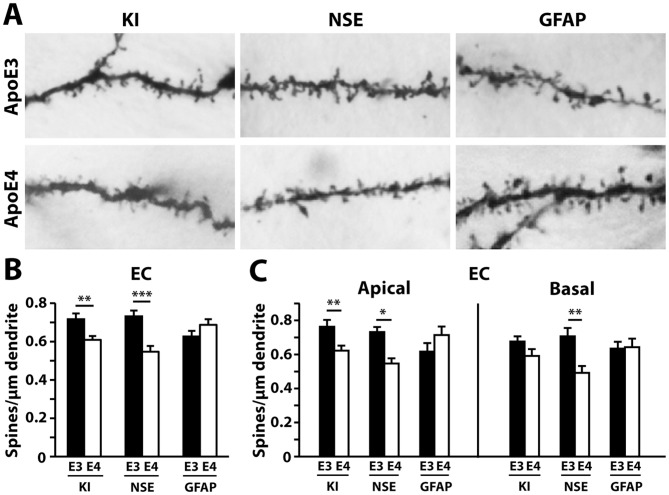
NSE-apoE4 and apoE4-KI, but not GFAP-apoE4, mice have reduced dendritic spine density in the entorhinal cortex at 19–21 months of age. (*A*) Representative images of dendrites with spines from the entorhinal cortex of NSE-apoE3, NSE-apoE4, apoE3-KI, apoE4-KI, GFAP-apoE3, or GFAP-apoE4 mouse brains. (*B*) Quantification of total spine density in the entorhinal cortex, combining AO+BS spines. (*C*) Number of AO and BS spines in the CA1. Values are mean ± SEM, n = 20–25 neurons from 3–5 mice for each group. *p<0.05, **p<0.01, ***p<0.001.

**Figure 6:**
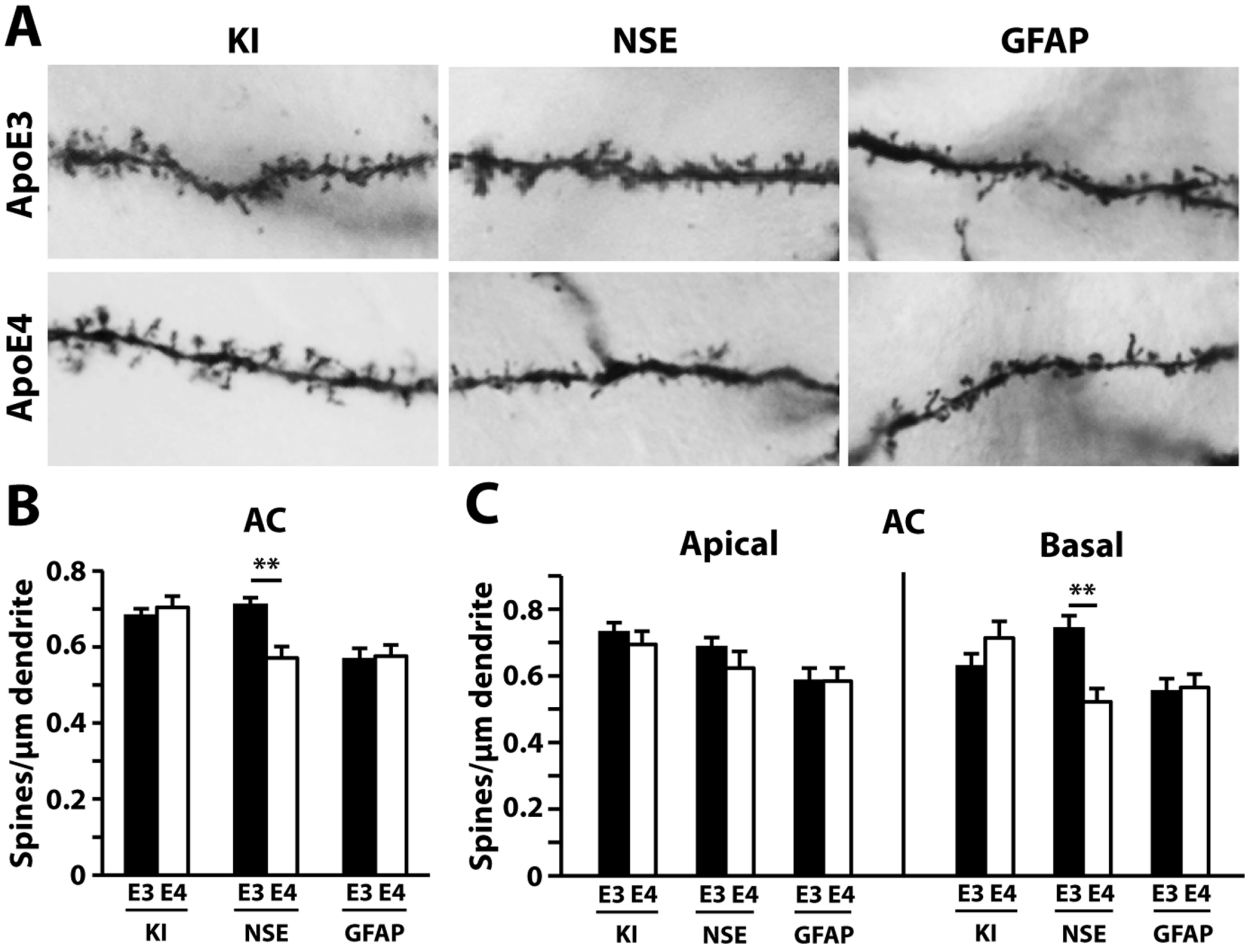
NSE-apoE4, but not GFAP-apoE4, mice have decreased spine density in the auditory cortex at 19–21 months of age. (*A*) Representative images of dendrites from the auditory cortex of NSE-apoE3, NSE-apoE4, apoE3-KI, apoE4-KI, GFAP-apoE3, or GFAP-apoE4 mouse brains. (*B*) Quantification of total spine density in the auditory cortex, combining AO+BS spines. (*C*) Number of AO and BS spines in the auditory cortex. Values are mean ± SEM, n = 20–24 neurons from 3–5 mice for each group. **p<0.01.

In the entorhinal cortex, similar decreases in total spine density were also evident in NSE-apoE4 (versus NSE-apoE3) and apoE4-KI (versus apoE3-KI) mice (Fig. 5A,B). Further classification of apical and basal spines revealed that in apoE4-KI mice, only the apical spine numbers were decreased, whereas NSE-apoE4 mice had fewer spines along both apical and basal dendrites (Fig. 5C). Astrocytic apoE expression did not affect total spine densities in either the apical or basal neuronal regions, as these densities were highly similar between GFAP-apoE4 and GFAP-apoE3 mice (Fig. 5A–C). One-way ANOVA revealed significant reductions in total, apical, and basal spines in NSE-apoE4 mice compared to GFAP-apoE4 mice (Fig. 5B,C). Finally, in the auditory cortex, NSE-apoE4 mice showed a significant decrease in spine density compared to NSE-apoE3 mice (Fig. 6A,B), particularly along basal shaft dendrites (Fig. 6C). Neither apoE4-KI nor GFAP-apoE4 mice had spine alterations in the auditory cortex compared to their apoE3 controls (Fig. 6A–C). One-way ANOVA revealed significant reductions in total and basal spines in NSE-apoE4 mice compared to apoE4-KI mice (Fig. 6B,C).

### The Cellular Source of apoE4 Determines its Effects on Dendritic Spine Morphology

**Figure 7:**
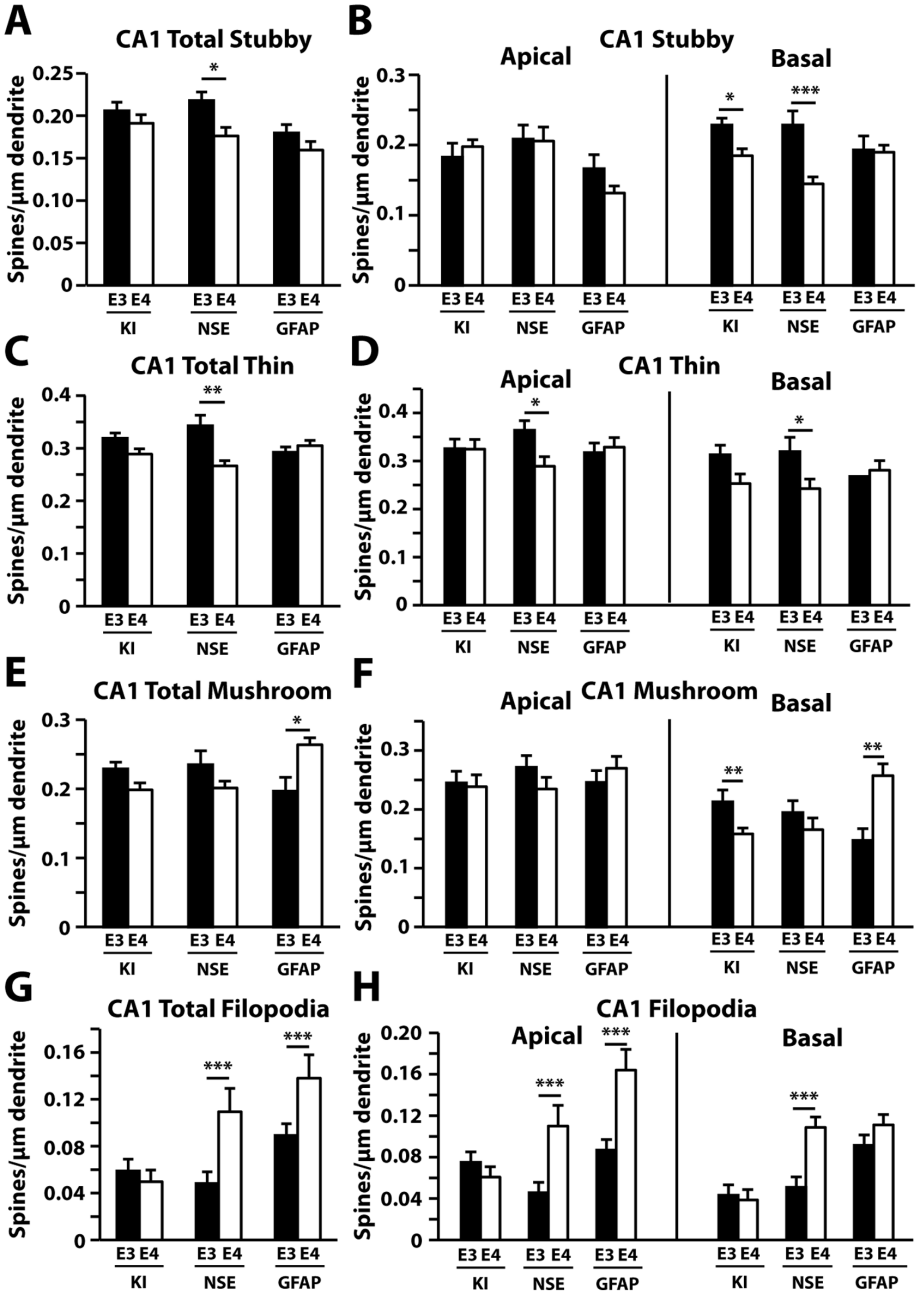
Quantification of spine subtypes in the CA1 region of the hippocampus in different apoE mouse models at 19–21 months of age. (*A–H*) Averaged densities of stubby (*A–B*), thin (*C–D*), mushroom (*E–F*), and filopodia (*G–H*) type protrusions in the CA1 region for all genotypes. Values are mean ± SEM, n = 20–33 neurons from 3–5 mice for each group. *p<0.05, **p<0.01, ***p<0.001.

Since dendritic spine morphology reflects synaptic function and plasticity [36], we also compared the numbers of spine subtypes in different brain regions of the apoE3 or apoE4 mice. Dendritic protrusions were classified into four categories based on the head size and neck length: stubby, thin, mushroom, and filopodia [31], [34]. All data were summarized in Table 1. In the CA1 region of the hippocampus, we observed decreases in the total number of stubby (Fig. 7A) and thin (Fig. 7C) spines in NSE-apoE4 compared to NSE-apoE3 mice. Selective examination of the basal dendrites revealed a significant drop in stubby spines in apoE4-KI mice as well as NSE-apoE4 mice compared to their apoE3 counterparts (Fig. 7B), whereas thin spines were only reduced in NSE-apoE4 mice compared to NSE-apoE3 mice, but were reduced in both apical and basal dendrites (Fig. 7D). ApoE4-KI mice had fewer basal mushroom spines compared to apoE3-KI mice (Fig. 7F), whereas GFAP-apoE4 mice had more mushroom spines than GFAP-apoE3 mice (Fig. 7E), and this difference was reflected in basal but not apical spine density (Fig. 7F). One-way ANOVA revealed significant reductions in total and basal mushroom spines in apoE4-KI and NSE-apoE4 mice compared to GFAP-apoE4 mice (Fig. 7E,F). Both NSE-apoE4 and GFAP-apoE4 mice had a greater number of filopodia compared to their apoE3 counterparts in the CA1 (Fig. 7G–H).

**Table 1:** Summary of the effects of apoE genotype on dendritic spine morphology in the CA1 subregion of the hippocampus, the entorhinal cortex, and the auditory cortex in different apoE mice at 19–21 months of age.

	CA1			Entorhinal Cortex			Auditory Cortex		
Spines Shape	Total	Apical	Basal	Total	Apical	Basal	Total	Apical	Basal
**Stubby**	NSE-E4< NSE-E3	No effect	E4-KI<E3-KINSE-E4< NSE-E3	No effect	No effect	No effect	No effect	No effect	No effect
**Thin**	NSE-E4< NSE-E3	NSE-E4< NSE-E3	NSE-E4< NSE-E3	E4-KI<E3-KI	E4-KI<E3-KI	No effect	No effect	No effect	No effect
**Mushroom**	GFAP-E4> GFAP-E3	No Effect	E4-KI<E3-KIGFAP-E4> GFAP-E3	NSE-E4< NSE-E3	No effect	NSE-E4< NSE-E3	NSE-E4< NSE-E3	No effect	NSE-E4< NSE-E3
**Filopodia**	NSE-E4> NSE-E3GFAP-E4> GFAP-E3	NSE-E4> NSE-E3GFAP-E4> GFAP-E3	NSE-E4> NSE-E3	No effect	No effect	No effect	GFAP-E4> GFAP-E3	GFAP-E4> GFAP-E3	GFAP-E4> GFAP-E3

**Figure 8:**
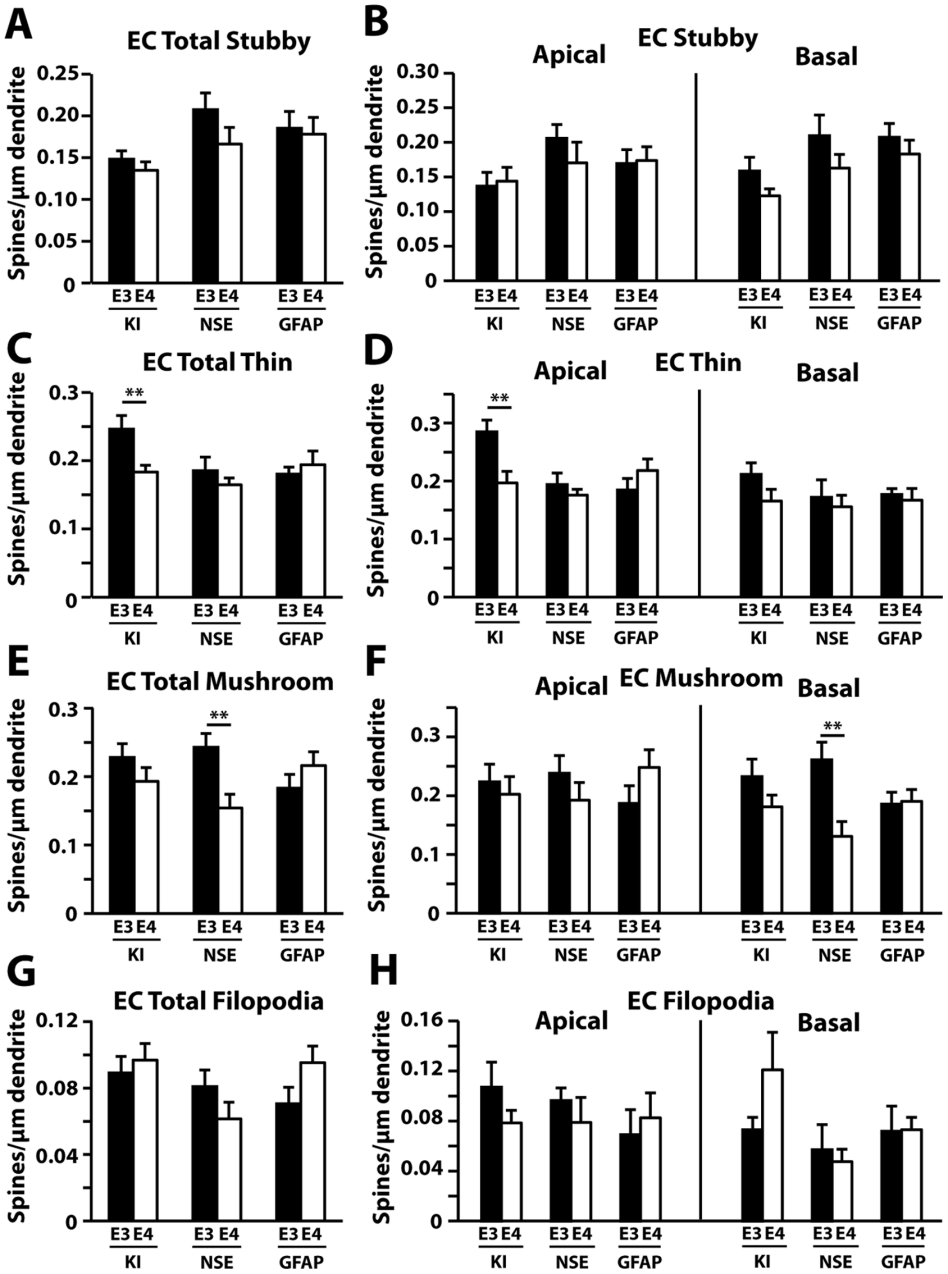
Quantification of spine subtypes in the entorhinal cortex (EC) in different apoE mouse models at 19–21 months of age. (*A–H*) Averaged densities of stubby (*A–B*), thin (*C–D*), mushroom (*E–F*), and filopodia (*G–H*) type protrusions in the entorhinal cortex for all genotypes. Values are mean ± SEM, n = 20–33 neurons from 3–5 mice for each group. **p<0.01.

**Figure 9:**
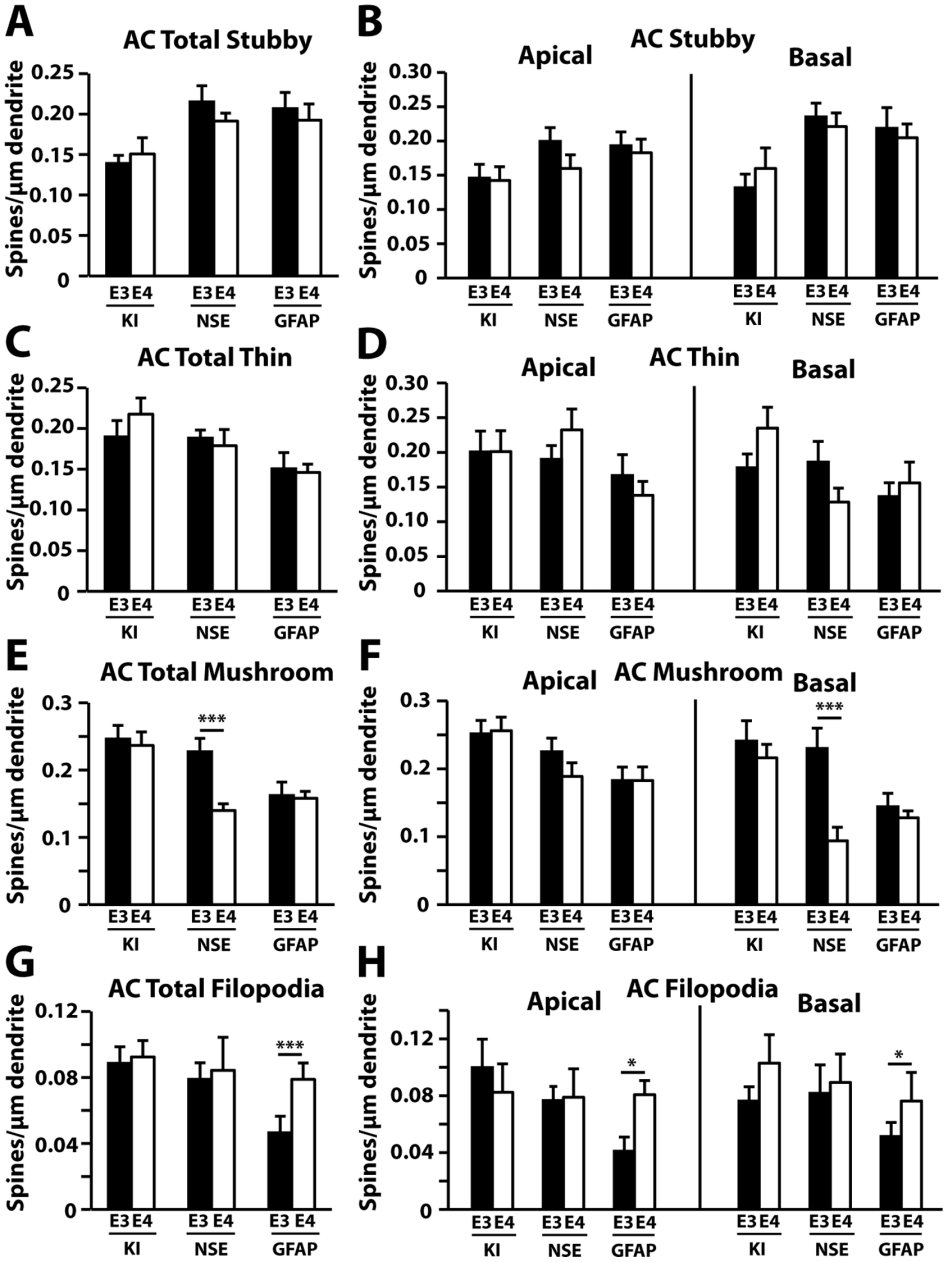
Quantification of spine subtypes in the auditory cortex (AC) in different apoE mouse models at 19–21 months of age. (*A–H*) Averaged densities of stubby (*A–B*), thin (*C–D*), mushroom (*E–F*), and filopodia (*G–H*) type protrusions in the auditory cortex for all genotypes. Values are mean ± SEM, n = 20–33 neurons from 3–5 mice for each group. *p<0.05, **p<0.01, ***p<0.001.

For details, see data presented in Figures 7, 8, and 9.

In the entorhinal cortex, thin spine density was decreased in apoE4-KI compared to apoE3-KI mice (Fig. 8C), which involved the apical, but not basal, spine subset (Fig. 8D). NSE-apoE4 mice had fewer total (Fig. 8E) and basal (Fig. 8F) mushroom spines compared to NSE-apoE3 mice in this region. One-way ANOVA revealed significant reductions in total and basal mushroom spines in NSE-apoE4 mice compared to GFAP-apoE4 mice (Fig. 8E,F). The densities of stubby spines (Fig. 8A–B) and filopodia (Fig. 8G–H) did not differ between any pairs in the entorhinal cortex. Finally, in the auditory cortex, NSE-apoE4 mice showed a reduction in total (Fig. 9E) and basal (Fig. 9F) mushroom spine densities compared to NSE-apoE3 mice. One-way ANOVA revealed significant reductions in total and basal mushroom spines in NSE-apoE4 and GFAP-apoE4 mice compared to apoE4-KI mice (Fig. 9E,F). The number of filopodia was higher in GFAP-apoE4 mice compared to GFAP-apoE3 mice (Fig. 9G–H). There were no significant differences in stubby (Fig. 9A–B) or thin (Fig. 9C–D) spine densities between any pairs in the auditory cortex.

### The Dendritic Spine Loss Occurs in NSE-apoE4 Mice at Young Ages

**Figure 10:**
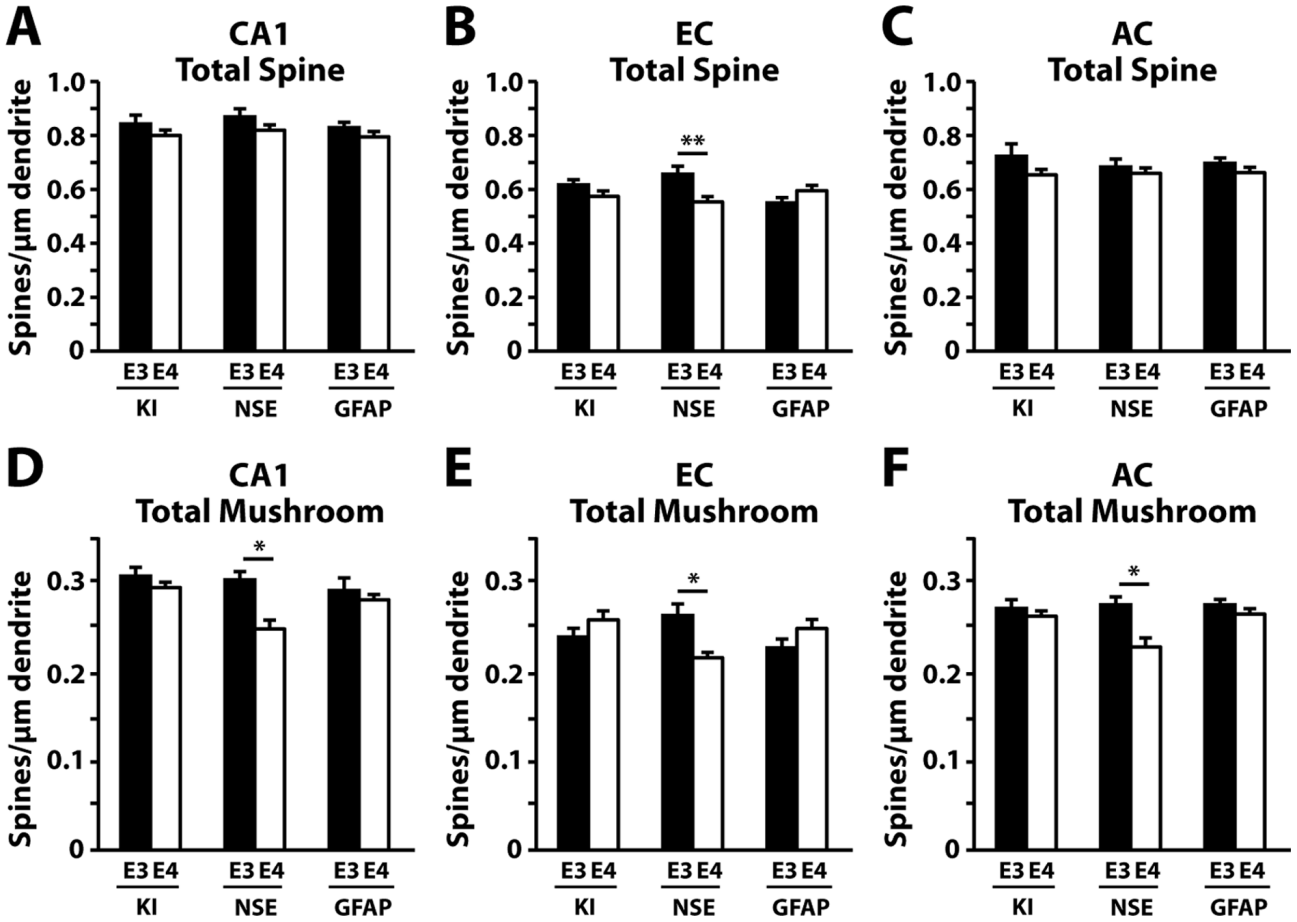
NSE-apoE4, but not apoE4-KI and GFAP-apoE4, mice have decreased total and mushroom spine densities at 7–8 months of age. (*A–C*) Quantification of total spine densities in the CA1 subregion of the hippocampus (*A*), the entorhinal cortex (*B*), and the auditory cortex (*C*). (*D–F*) Averaged densities of mushroom spines in the CA1 subregion of the hippocampus (*D*), the entorhinal cortex (*E*), and the auditory cortex (*F*). Values are mean ± SEM, n = 27–29 neurons from 4–5 mice for each group. *p<0.05, **p<0.01.

We previously reported that NSE-apoE4 mice had learning and memory deficits at 7–8 months of age [13], [15], [16], whereas apoE4-KI and GFAP-apoE4 mice did not have similar deficits at these ages [17], [18]. To explore the potential relationship between apoE4-induced dendritic spine loss and behavioral deficits, we also assessed the dendritic arborization and spine density and morphology in different brain regions of the apoE3 or apoE4 mice at 7–8 months of age. Although apoE4 had no significant effects on dendritic arborization in all three models of mice at these young ages (data not shown), it significantly decreased total spine density in the entorhinal cortex of NSE-apoE4 mice compared to NSE-apoE3 mice (Fig. 10B). ApoE4 also significantly decreased total mushroom spines in the CA1 subregion of the hippocampus, the entorhinal cortex, and the auditory cortex of NSE-apoE4 mice compared to NSE-apoE3 mice (Fig. 10D–F). One-way ANOVA revealed significant reductions in total mushroom spines in NSE-apoE4 mice compared to apoE4-KI and GFAP-apoE4 mice in the CA1, the entorhinal cortex, and the auditory cortex (Fig. 10D–F). ApoE4-KI and GFAP-apoE4 mice had no differences in their spine densities in all three regions of the brain compared to their respective apoE3-expressing counterparts (Fig. 10D–F).

## Discussion

In this study, we used mice expressing human apoE to determine whether the effects of apoE on dendritic morphology depend, at least in part, on the cell type in which it is synthesized. Indeed, in female apoE mice at 19–21 months of age, we report cellular source-dependent effects of apoE4 on dendrite arborization as well as spine density and morphology *in vivo*. Whereas NSE-apoE4 and apoE4-KI mice show dendritic arborization deficits and/or spine loss in the hippocampus and different regions of the cortex compared to their respective apoE3 counterparts, GFAP-apoE4 mice do not have such impairments. Furthermore, of the mouse models investigated, NSE-apoE4 mice had the most severe and widespread morphological deficits, followed by apoE4-KI mice. The loss of dendritic spines, especially mushroom spines, occurred in NSE-apoE4 mice as early as 7–8 months of age. These data suggest that neuron-derived apoE4 is especially neurotoxic, with profound effects on dendrite arborization, spine density, and spine morphology, while astrocyte-derived apoE4 does not have these detrimental effects.

### The Effect of apoE4’s Cellular Source on Dendrite Development

Previous studies have shown that apoE3 promotes neurite outgrowth and dendritic arborization in neuronal and brain slice cultures, whereas apoE4 inhibits these processes [21], [22], [37]. Neurite degeneration has been observed in transgenic mouse models of tauopathy [38] and in post-mortem AD patient brains [39], suggesting that dendrite abnormalities are an important component of AD-related neuropathology. Furthermore, it has been reported that neuron-derived apoE4 has a more detrimental effect on dendritic integrity compared to astrocytic apoE4 in response to excitotoxic injury, as evidenced by reduced MAP-2 immunostaining in NSE-apoE4, but not in GFAP-apoE4, transgenic mice [15]. In this study, we show that mice expressing apoE3 specifically in neurons have an increased number of total dendrites in both the CA1 and entorhinal cortex compared to neuronal apoE4–expressing mice, and the dendrites of NSE-apoE4 mice are shorter than those of NSE-apoE3 mice in these brain regions. We postulate that the toxicity of apoE4 produced by neurons contributes to the dendritic arborization impairments during aging and in response to brain injuries, while neuronal apoE3 promotes dendrite development.

In our experiments, neither apoE4-KI nor GFAP-apoE4 mice showed differences in dendritic arborization in the CA1 or entorhinal cortex compared to their respective apoE3 counterparts, while in the auditory cortex, GFAP-apoE4 mice had longer and more dendrites than GFAP-apoE3 mice. In contrast, previous studies described a decrease in dendrite length and number of GFAP-apoE4 mice compared to GFAP-apoE3 mice [23]. These discrepancies might be due to several factors, including the age of the mice and the brain areas investigated. For instance, we examined the CA1 region of the hippocampus, the entorhinal cortex, and the auditory cortex at 7–8 and 19–21 months of age, while the previous study focused on the dentate gyrus of mice at 12 and 24 months of age [23]. Differences in spine labeling and quantification methods between our study (Golgi staining of whole brains *in vivo*) and Ji *et al* (gene gun–labeled cultured brain slices *ex vivo*) might have also contributed to these outcomes. Importantly, our results indicate that neuronal apoE4 is detrimental to dendritic arborization in brain regions that are affected during the early stages of AD [40], whereas astrocyte-derived apoE4 is not associated with such impairments in these regions.

### Cellular Source-dependent Effects of apoE4 on Dendritic Spine Development and Morphology

The loss of dendritic spines and synapses is the best pathological correlate for cognitive impairments in AD [19], [41]. We found that total spine density is reduced in the CA1 region of the hippocampus, entorhinal cortex, and auditory cortex of NSE-apoE4 mice compared to NSE-apoE3 mice at 19–21 months of age. Importantly, the total spine loss occurs in the entorhinal cortex of NSE-apoE4 mice as early as 7–8 months of age. ApoE4-KI mice also had fewer total spines in the CA1 and entorhinal cortex at old (19–21 months) but not young (7–8 months) ages. These results support previous findings that apoE4 is detrimental to spine development *in vitro* and *in vivo* [24], [25], [26]. However, our study is the first to show a cellular source-dependent effect of apoE4 on spine numbers, as GFAP-apoE4 mice, which express apoE4 exclusively in astrocytes, did not have decreased spine density in any of the brain regions analyzed compared to GFAP-apoE3 mice at both young and old ages. We previously reported that NSE-apoE4 mice start to develop spatial learning and memory deficits at 6–8 months of age [16], while apoE4-KI mice do not develop these deficits until 16–17 months of age [17]. In contrast, GFAP-apoE4 mice do not show spatial learning and memory deficits even at the old ages [18]. Thus, the cellular source-dependent detrimental effects of apoE4 on dendritic spines correlate temporally with apoE4-induced spatial learning and memory deficits in mice.

Spines form a variety of sizes and shapes that correlate with their synaptic plasticity and strength. Whereas thin “learning” spines are highly plastic and are subject to rapid formation and elimination, mushroom spines are more stable and are thus termed “memory” spines [36], [42]. Previous studies found that apoE4-KI mice have shorter spines in cortical layers II/III compared to apoE3-KI mice [26], but these experiments did not address the potential effects of apoE4’s cellular source on the distribution of spine subtypes. We found that the source of apoE4 not only affected spine density in several brain regions, but also influenced spine morphology. For instance, whereas NSE-apoE4 mice had fewer thin (old mice) and mushroom (both young and old mice) spines in the CA1 and entorhinal cortex compared to NSE-apoE3 mice, GFAP-apoE4 mice did not show a reduction in the densities of these spine subtypes (both young and old mice). These data suggest that neuronal apoE4 inhibits the development of specific subsets of spines that are associated with learning and memory, whereas astrocytic apoE4 does not impair these subtypes.

Interestingly, apoE4-KI mice, in which apoE4 is primarily expressed in astrocytes, also had decreased spine density and impairment of specific subsets of spines, although to a lesser extent than NSE-apoE4 mice. Since GFAP-apoE4 mice, in which apoE4 is exclusively expressed in astrocytes, did not have such impairments, the detrimental effects of apoE4 on dendritic spines in apoE4-KI mice is likely caused by non-astrocytic sources of apoE4. One possibility is that, during aging, apoE4 expression is turned on in neurons as proposed previously [3], [4], [10], [11], leading to its detrimental effect on neuronal spine development and morphology. Alternatively, apoE4 made in cells other than neurons and astrocytes might contribute to such detrimental effects.

There are a number of potential mechanisms through which neuronal apoE4 might exert its inhibitory effects on spine density and morphology. Neuronal apoE4 is susceptible to proteolytic cleavage, producing C-terminally truncated fragments that are associated with increased tau phosphorylation [3], [4], [13], [14]. The accumulation of hyperphosphorylated tau in spines has been linked to disrupted synaptic function through the impairment of glutamate receptor trafficking and synaptic anchoring [43]. Thus, neuronal apoE4 might reduce spine numbers by enhancing tau phosphorylation and localization into spines. Astrocyte-derived apoE4, on the other hand, is not associated with apoE fragmentation and tau phosphorylation [13], which might explain why it does not inhibit spine density compared to astrocytic apoE3. Neuronal apoE4 and its fragments also impair mitochondrial integrity, dynamics, and function [3], [12], [44]. Normal mitochondrial dynamics and function are essential for the morphogenesis and plasticity of spines and synapses [45]. Recently, an inherited variable poly-T repeat genotype of mitochondrial protein TOMM40 has been suggested to be associated with the age-of-onset of AD and cognitive aging [28], [46]. It has also been suggested that apoE4 interacts with TOMM40 to cause mitochondrial impairment and neurotoxicity [28]. In this regard, apoE4 or its fragments might interact with TOMM40 on the mitochondrial outer membrane, causing the release of cytochrome C from damaged mitochondria with subsequent apoptosis, which could be a therapeutic target for AD [28]. In addition, apoE4 also shows cellular source-dependent differences in its modulation of excitotoxic responses. Whereas both neuronal and astrocytic apoE3 protected dendrites and synapses against excitotoxic injury in the neocortex and hippocampus, apoE4 was excitoprotective only when produced by astrocytes [15]. The loss of this excitoprotective function of neuronal apoE4 is likewise due to its susceptibility to proteolysis [15].

In conclusion, our study demonstrates for the first time that the cellular source of apoE determines its effects on dendritic arborization, spine density, and spine morphology. Neuron-derived apoE4 has detrimental effects on dendritic arborization and spine development, whereas astrocytic apoE4 does not. These studies should further our understanding of the functional differences of apoE4 derived from different cellular sources in the brain and provide insights for drug development targeting apoE4’s detrimental effects.

## References

[1] Mahley RW, Weisgraber KH, Huang Y (2006) Apolipoprotein E4: a causative factor and therapeutic target in neuropathology, including Alzheimer’s disease. Proc Natl Acad Sci U S A 103: 5644–5651. 16567625 10.1073/pnas.0600549103 PMC1414631

[2] Kim J, Basak JM, Holtzman DM (2009) The role of apolipoprotein in Alzheimer’s disease. Neuron 63: 287–303. 19679070 10.1016/j.neuron.2009.06.026 PMC3044446

[3] Huang YH (2010) Aβ-independent roles of apolipoprotein E4 in the pathogenesis of Alzheimer’s disease. Trends Mol Med 16: 287–294. 20537952 10.1016/j.molmed.2010.04.004

[4] Huang Y, Mucke L (2012) Alzheimer mechanisms and therapeutic strategies. Cell 148: 1204–1222. 22424230 10.1016/j.cell.2012.02.040 PMC3319071

[5] Strittmatter WJ, Saunders AM, Schmechel D, Pericak-Vance M, Enghild J, et al (1993) Apolipoprotein E: high-avidity binding to beta-amyloid and increased frequency of type 4 allele in late-onset familial Alzheimer disease. Proc Natl Acad Sci U S A 90: 1977–1981. 8446617 10.1073/pnas.90.5.1977 PMC46003

[6] Roses AD (1996) Apolipoprotein E alleles as risk factors in Alzheimer’s disease. Annu Rev Med 47: 387–400. 8712790 10.1146/annurev.med.47.1.387

[7] Corder EH, Saunders AM, Risch NJ, Strittmatter WJ, Schmechel DE, et al (1993) Gene dose of apolipoprotein E type 4 allele and the risk of Alzheimer’s disease in late onset families. Science 261: 921–923. 8346443 10.1126/science.8346443

[8] Farrer LA, Cupples LA, Haines JL, Hyman B, Kukull WA, et al (1997) Effects of age, sex, and ethnicity on the association between apolipoprotein E genotype and Alzheimer disease. A meta-analysis. JAMA 278: 1349–1356. 9343467

[9] Boschert U, Merlo-Pich E, Higgins G, Roses AD, Catsicas S (1999) Apolipoprotein E expression by nerons surviving excitotoxic stress. Neurobiol Dis 6: 508–514. 10600406 10.1006/nbdi.1999.0251

[10] Xu Q, Bernardo A, Walker D, Kanegawa T, Mahley RW, et al (2006) Profile and regulation of apolipoprotein E (ApoE) expression in the CNS of mice with targeting of green fluorescent protein gene to the ApoE locus. J Neurosci 26: 4985–4994. 16687490 10.1523/JNEUROSCI.5476-05.2006 PMC6674234

[11] Xu Q, Walker D, Bernardo A, Brodbeck J, Balestra ME, et al (2008) Intron-3 retention/splicing controls neuronal expression of apolipoprotein E in the CNS. J Neurosci 28: 1452–1459. 18256266 10.1523/JNEUROSCI.3253-07.2008 PMC6671590

[12] Chang S, Ma TR, Miranda RD, BAlestra ME, Mahley RW, Huang Y (2005) Lipid- and receptor-binding regions of apolipoprotein E4 fragments act in concert to cause mitochondrial dysfunction and neurotoxicity. Proc Natl Acad Sci USA. 102: 18694–18699. 10.1073/pnas.0508254102 PMC1311737 16344479

[13] Brecht WJ, Harris FM, Chang S, Tesseur I, Yu GQ, et al (2004) Neuron-specific apolipoprotein E4 proteolysis is associated with increased tau phosphorylation in brains of transgenic mice. J Neurosci 24: 2527–2534. 15014128 10.1523/JNEUROSCI.4315-03.2004 PMC6729489

[14] Harris FM, Brecht WJ, Xu Q, Tesseur I, Kekonius L, et al (2003) Carboxyl-terminal-truncated apolipoprotein E4 causes Alzheimer’s disease-like neurodegeneration and behavioral deficits in transgenic mice. Proc Natl Acad Sci U S A 100: 10966–10971. 12939405 10.1073/pnas.1434398100 PMC196910

[15] Buttini M, Masliah E, Yu GQ, Palop JJ, Chang S, et al (2010) Cellular source of apolipoprotein E4 determines neuronal susceptibility to excitotoxic injury in transgenic mice. Am J Pathol 177: 563–569. 20595630 10.2353/ajpath.2010.090973 PMC2913361

[16] Raber J, Wong D, Buttini M, Orth M, Bellosta S, et al (1998) Isoform-specific effects of human apolipoprotein E on brain function revealed in apoE knockout mice: increased susceptibility of females. Proc Natl Acad Sci U S A 95: 10914–10919. 9724804 10.1073/pnas.95.18.10914 PMC27995

[17] Andrews-Zwilling Y, Bien-Ly N, Xu Q, Li G, Bernardo A, et al (2010) Apolipoprotein E4 causes age-and Tau-dependent impairment of GABAergice interneurons, leading to learning and memory deficits in mice. J Neurosci 30: 13707–13717. 20943911 10.1523/JNEUROSCI.4040-10.2010 PMC2988475

[18] Hartman RE, Wozniak DF, Nardi A, Olney JW, Sartorius L, et al (2001) Behavioral phenotyping of GFAP-apoE3 and -apoE4 transgenic mice: apoE4 mice show profound working memory impairments in the absence of Alzheimer’s-like neuropathology. Exp Neurol 170: 326–344. 11476599 10.1006/exnr.2001.7715

[19] Masliah E, Mallory M, Alford M, DeTeresa R, Hansen LA, et al (2001) Altered expression of synaptic proteins occurs early during progression of Alzheimer’s disease. Neurology 56: 127–129. 11148253 10.1212/wnl.56.1.127

[20] Baloyannis SJ (2009) Dendritic pathology in Alzheimer’s disease. J Neurol Sci 283: 153–157. 19296966 10.1016/j.jns.2009.02.370

[21] Nathan BP, Jiang Y, Wong GK, Shen F, Brewer GJ, et al (2002) Apolipoprotein E4 inhibits, and apolipoprotein E3 promotes neurite outgrowth in cultured adult mouse cortical neurons through the low-density lipoprotein receptor-related protein. Brain Res 928: 96–105. 11844476 10.1016/s0006-8993(01)03367-4

[22] Bellosta S, Nathan BP, Orth M, Dong LM, Mahley RW, et al (1995) Stable expression and secretion of apolipoproteins E3 and E4 in mouse neuroblastoma cell produces differential effects on neurite outgrowth. J Biol Chem 270: 27063–27071. 7592957 10.1074/jbc.270.45.27063

[23] Ji Y, Gong Y, Gan W, Beach T, Holtzman DM, et al (2003) Apolipoprotein E isoform-specific regulation of dendritic spine morphology in apolipoprotein E transgenic mice and Alzheimer’s disease patients. Neuroscience 122: 305–315. 14614898 10.1016/j.neuroscience.2003.08.007

[24] Brodbeck J, Balestra ME, Saunders AM, Roses AD, Mahley RW, et al (2008) Rosiglitazone increases dendritic spine density and rescues spine loss caused by apolipoprotein E4 in primary cortical neurons. Proc Natl Acad Sci U S A 105: 1343–1346. 18212130 10.1073/pnas.0709906104 PMC2234140

[25] Brodbeck J, McGuire J, Liu Z, Meyer-Franke A, Balestra ME, et al (2011) Structure-dependent impairment of intracellular apolipoprotein E4 trafficking and its detrimental effects are rescued by small-molecule structure correctors. J Biol Chem 286: 17217–17226. 21454574 10.1074/jbc.M110.217380 PMC3089564

[26] Dumanis SB, Tesoriero JA, Babus LW, Nguyen MT, Trotter JH, et al (2009) ApoE4 decreases spine density and dendritic complexity in cortical neurons in vivo. J Neurosci 29: 15317–15322. 19955384 10.1523/JNEUROSCI.4026-09.2009 PMC2846754

[27] Roses AD, Saunders AM (2006) Perspective on a pathogenesis and treatment of Alzheimer’s disease. Alzheimers Dement 2: 59–70. 19595857 10.1016/j.jalz.2005.12.001

[28] Roses AD (2010) An inherited variable Ploy-T repeat genotype in TOMM40 in Alzheimer Disease. Arch Neurol 67: 536–541. 20457951 10.1001/archneurol.2010.88 PMC3140162

[29] Buttini M, Orth M, Bellosta S, Akeefe H, Pitas RE, et al (1999) Expression of human apolipoprotein E3 or E4 in the brains of *Apoe* ^−/−^ mice: isoform-specific effects on neurodegeneration. J Neurosci 19: 4867–4880. 10366621 10.1523/JNEUROSCI.19-12-04867.1999 PMC6782676

[30] Sullivan PM, Mace BE, Maeda N, Schmechel DE (2004) Marked regional differences of brain human apolipoprotein E expression in targeted replacement mice. Neuroscience 124: 725–733. 15026113 10.1016/j.neuroscience.2003.10.011

[31] Jain S, Yoon SY, Zhu L, Brodbeck J, Dai J, et al (2012) Arf4 determines dentate gyrus-mediated pattern separation by regulating dendritic spine development. PLoS ONE 7: e46340. 23050017 10.1371/journal.pone.0046340 PMC3457985

[32] Pool M, Thiemann J, Bar-Or A, Fournier AE (2008) NeuriteTracer: a novel ImageJ plugin for automated quantification of neurite outgrowth. J Neurosci Methods 169: 134–139. 10.1016/j.jneumeth.2007.08.029 17936365

[33] Vanderklish PW, Edelman GE (2002) Dendritic spines elongate after stimulation of group 1 metabotropic glutamate receptors in cultured hippocampal neurons. Proc Natl Acad Sci U S A 99: 1639–1644. 11818568 10.1073/pnas.032681099 PMC122243

[34] Peebles CL, Yoo J, Thwin MT, Palop JJ, Noebels JL, et al (2010) Arc regulates spine morphology and maintains network stability in vivo. Proc Natl Acad Sci U S A 107: 18173–18178. 20921410 10.1073/pnas.1006546107 PMC2964216

[35] Raber J, Wong D, Yu GQ, Buttini M, Mahley RW, et al (2000) Apolipoprotein E and cognitive performance. Nature 404: 352–354. 10.1038/35006165 10746713

[36] Tada T, Sheng M (2006) Molecular mechanisms of dendritic spine morphogenesis. Curr Opin Neurobiol 16: 95–101. 16361095 10.1016/j.conb.2005.12.001

[37] Teter B, Xu PT, Gilbert JR, Roses AD, Galasko D, et al (1999) Human apolipoprotein E isoform-specific differences in neuronal sprouting in organotypic hippocampal culture. J Neurochem 73: 2613–2616. 10582625 10.1046/j.1471-4159.1999.0732613.x

[38] Jaworski T, Lechat B, Demedts D, Gielis L, Devijver H, et al (2011) Dendritic degeneration, neurovascular defects, and inflammation precede neuronal loss in a mouse model for tau-mediated neurodegeneration. Am J Pathol 179: 2001–2015. 21839061 10.1016/j.ajpath.2011.06.025 PMC3181369

[39] Einstein G, Buranosky R, Crain BJ (1994) Dendritic pathology of granule cells in Alzheimer’s disease is unrelated to neuritic plaques. J Neurosci 14: 5077–5088. 8046469 10.1523/JNEUROSCI.14-08-05077.1994 PMC6577187

[40] Von Gunten A, Kovari E, Bussiere T, Rivara CB, Gold G, et al (2006) Cognitive impact of neuronal pathology in the entorhinal cortex and CA1 field in Alzheimer’s disease. Neurobiol Aging 27: 270–277. 16399212 10.1016/j.neurobiolaging.2005.02.008

[41] DeKosky ST, Scheff SW (1990) Synapse loss in frontal cortex biopsies in Alzheimer’s disease: correlation with cognitive severity. Ann Neurol 27: 457–464. 2360787 10.1002/ana.410270502

[42] Bourne J, Harris KM (2007) Do thin spines learn to be mushroom spines that remember? Curr Opin Neurobiol 17: 381–386. 17498943 10.1016/j.conb.2007.04.009

[43] Hoover BR, Reed MN, Su J, Penrod RD, Kotilinek LA, et al (2010) Tau mislocalization to dendritic spines mediates synaptic dysfunction independently of neurodegeneration. Neuron 68: 1067–1081. 21172610 10.1016/j.neuron.2010.11.030 PMC3026458

[44] Chen HK, Liu Z, Meyer-Franke A, Brodbeck J, Miranda RD, et al (2012) Small molecule structure correctors abolish detrimental effects of apolipoprotein E4 in cultured neurons. J Biol Chem 287: 5253–5266. 22158868 10.1074/jbc.M111.276162 PMC3285306

[45] Li Z, Okamoto K, Hayashi Y, Sheng M (2004) The importance of dendritic mitochondria in the morphogenesis and plasticity of spines and synapses. Cell 119: 873–887. 15607982 10.1016/j.cell.2004.11.003

[46] Caselli RJ, Dueck AC, Huentelman MJ, Lutz MW, Saunders AM, Reiman EM, et al (2012) Longitudinal modeling of cognitive aging and the TOMM40 effect. Alzheimers Dement 8: 490–495. 23102119 10.1016/j.jalz.2011.11.006 PMC3483561

